# GM-CSF production by immune cells in steady state and autoimmune neuroinflammation mapped using fate reporting mice

**DOI:** 10.3389/fimmu.2025.1617074

**Published:** 2025-08-19

**Authors:** Gholamreza Azizi, Javad Rasouli, Hamed Naziri, Michael V. Gonzalez, James Garifallou, Guang-Xian Zhang, Bogoljub Ciric, Abdolmohamad Rostami

**Affiliations:** ^1^ Department of Neurology, Thomas Jefferson University, Philadelphia, PA, United States; ^2^ Center for Cytokine Storm Treatment and Laboratory, University of Pennsylvania, Philadelphia, PA, United States; ^3^ Division of Neonatology, Children’s Hospital of Philadelphia, Philadelphia, PA, United States

**Keywords:** GM-CSF, CD4+ T cells, CXCR6, fate reporting, neuroinflammation, experimental autoimmune encephalomyelitis

## Abstract

**Introduction:**

GM-CSF is a pro-inflammatory cytokine that promotes an inflammatory phenotype in myeloid cells. The extent and pattern of GM-CSF expression in immune cells have not been fully elucidated. Our goal was to advance this topic using novel GM-CSF reporter/fate reporter transgenic mice.

**Methods:**

We tracked ongoing and past GM-CSF expression in various immune cells from multiple organs, in steady-state and autoimmune inflammation of the central nervous system (CNS).

**Results:**

The GM-CSF expression patterns varied by cell type and organ, with CD4^+^, CD8^+^, and CD11b^+^ cells being the main producers. GM-CSF expression was transient and seemingly permanently lost in most cells over time. In a mouse model of CNS autoimmunity, effector memory CD4^+^ T cells were the dominant GM-CSF source in the CNS. A large proportion of CD4^+^ T cells that expressed GM-CSF also expressed CXCR6, but this chemokine receptor did not play a main role in the CNS autoimmunity. Transcriptomic analysis showed notably distinct gene expression profiles between effector memory CD4^+^ T cells that did and did not express GM-CSF.

**Discussion:**

These findings identified distinct GM-CSF cellular sources across organs, highlighting the transient nature of GM-CSF expression and the correlation between its expression and the overall phenotype of effector memory CD4^+^ T cells.

## 1 Introduction

Granulocyte macrophage-colony stimulating factor (GM-CSF, also known as CSF2) is a hematopoietic growth factor and immune modulator that promotes the proliferation and differentiation of bone marrow (BM) progenitor cells into myeloid lineages (1, 2). GM-CSF heteromeric cell-surface receptor, comprising GM-CSFRα and GM-CSFRβc subunits, mediates its biological effects and is expressed on multiple cell types, including monocytes, macrophages, neutrophils, endothelial cells, and alveolar epithelial cells (3–5). While GM-CSF is typically expressed at low levels in healthy tissues (6) its production increases during inflammation and autoimmune diseases (5, 7–9).

GM-CSF is primarily produced by lymphoid and myeloid cells, particularly in response to inflammatory stimuli (8, 10). Among these cell types, T cells are recognized as likely the most relevant cellular source of GM-CSF in the pathogenesis of multiple sclerosis (MS) (11, 12). Although the contribution of GM-CSF to the encephalitogenicity of CD8^+^ T cells remains unknown (12, 13), CD4^+^ T cells are viewed as the predominant source of GM-CSF in neuroinflammation (8). Initially, Th1 and Th17 cells were thought to be the major producers of GM-CSF, playing a crucial role in the pathogenesis of experimental autoimmune encephalomyelitis (EAE) (11, 14, 15), an animal model of MS. However, recent studies identified a distinct GM-CSF-producing T helper (Th) subset, ThGM, that is probably involved in EAE development (8, 10, 16). MS patients exhibit an expansion of GM-CSF-secreting CD4^+^ T cells, indicating their pathogenic role in neuroinflammation (12, 17). Several non-T cell types also produce GM-CSF, contributing to inflammatory responses. These include B cells, innate lymphoid cells (ILCs), and resident tissue cells such as endothelial cells, fibroblasts, and epithelial cells (17–19). Collectively, these findings underscore the diverse cellular sources of GM-CSF in shaping inflammatory environments across different disease contexts.

GM-CSF expression *in vivo* has been explored only partially (10). To address this gap, we designed our study using a fate reporter system to answer the following key questions: 1. Which immune cells express GM-CSF, and how does its expression vary across tissues in steady state and inflammation? 2. Is the capacity for GM-CSF expression by CD4^+^ T cells a stable or transient feature? 3. Does a history of GM-CSF expression by CD4^+^ T cells correlate with their phenotype and/or transcriptional profile? 4. What surface markers or transcriptional signatures are associated with GM-CSF-producing T cells, and do these suggest tissue residency or functional specialization? 5. Which CD4^+^ T cell subsets produce GM-CSF during neuroinflammation, and how does this change over the disease course?

We characterized GM-CSF expression in both steady state and neuroinflammation, focusing on its production by CD4^+^ T cell subsets and other tissue-resident immune cells. Our findings highlight effector memory CD4^+^ T cells as the primary source of GM-CSF during neuroinflammation. Additionally, we observed that GM-CSF-expressing T cells co-express CXCR6, suggesting a potential link between GM-CSF production and tissue residency. Notably, there is a strong correlation between the history of GM-CSF expression by CD4^+^ T cells and their overall phenotype, as effector memory CD4^+^ T cells that have previously expressed GM-CSF and those that never did have vastly different transcriptomes. This indicates that factors driving/regulating GM-CSF expression by CD4^+^ T cells have additional widespread effects. Furthermore, our results show that GM-CSF expression in CD4^+^ T cells is transient and seemingly ceases permanently in most of the cells that express GM-CSF.

## 2 Materials and methods

### 2.1 Mice

The GM-CSF reporter (*Gr*) transgenic mouse line was generated in-house. *Gr* mice carry a transgenic GM-CSF (*Csf2*) allele that enables normal GM-CSF expression, along with iCre and blue fluorescent protein (BFP) expression (20). To create a GM-CSF reporter/fate reporter (*Gr/fr*) double transgenic line, we crossed *Gr* mice with Rosa26eYFP mice (The Jackson Laboratory). *Gr/fr* mice enable lineage tracing through iCre-induced continuous yellow fluorescent protein (YFP) expression (**Supplementary Figure S1**). Thus, cells with ongoing GM-CSF expression are BFP^+^ (reporting function), while cells that previously expressed GM-CSF are permanently YFP^+^ (fate reporting function) (8, 20). Since we stained for multiple cytokines in parallel, we opted to stain cells with anti-GM-CSF monoclonal antibody (mAb) instead of solely relying on BFP expression to identify GM-CSF-expressing cells. Nonetheless, in some experiments, we relied on BFP expressions to identify GM-CSF^+^ cells, and **Supplementary Figure S2A** demonstrates an excellent correlation between BFP and GM-CSF expression.

*Cxcr3* ^-^/^-^ and *Cxcr6* ^-^/^-^ mouse lines on the C57BL/6 background and wild-type (WT) C57BL/6 (B6) mice were purchased from the Jackson Laboratory.

### 2.2 Mononuclear cell isolation

Mice were anesthetized using a ketamine-xylazine cocktail consisting of ketamine (100 mg/kg) and xylazine (10 mg/kg) administered via intraperitoneal injection. After blood collection, mice were perfused through the left ventricle with cold PBS. Then, inguinal lymph nodes (LNs), spleen, BM, and thymus were collected and mechanically dissociated. The central nervous system (CNS), liver, small intestine (SI), and lung were collected and digested with DNase (50 µg/mL; Stem Cell) and Liberase (500 µg/mL for CNS; 200 µg/mL for liver, and SI; 100 µg/mL for lung; Sigma-Aldrich), in RPMI for 30 min at 37°C, followed by mechanical dissociation. The debris removal solution (Miltenyi Biotec) efficiently removed cell debris from the liver, and lungs. For the CNS, myelin was removed using a 40% Percoll (Cytiva) gradient at 2000 RPM for 30 min at 24°C. SI cells were suspended in a 40% Percoll (Cytiva) overlayed on a 70% Percoll and spun at 2000 RPM for 25 min at 24°C. Mononuclear cells (MNCs) were collected from the interface between Percoll layers. Peripheral blood mononuclear cells (PBMCs) were isolated from whole blood by Ficoll density gradient centrifugation.

### 2.3 Flow cytometry and intracellular staining

Cells were activated with PMA (50 ng/ml; Sigma-Aldrich), Ionomycin (500 ng/ml; Sigma-Aldrich), and GolgiPlug (1 µg/ml; BD Biosciences) at 37°C for 4–6 h. Cells were washed, and anti-CD16/32 Ab (eBioscience) was used to block the non-specific binding of Abs to Fc receptors on myeloid cells. The cells were then stained with Abs for surface markers (**Supplementary Table S1**). After fixation/permeabilization with Caltag Fix/Perm reagents (Invitrogen), cells were stained with Abs to detect intracellular markers (**Supplementary Table S1**). Data was acquired on FACSAria Fusion II (BD Biosciences) and analyzed by FlowJo software (TreeStar).

### 2.4 MHC tetramer staining

MNCs were isolated from the spleen, liver, and lung of myelin oligodendrocyte glycoprotein (MOG)_35-55_-immunized mice at 8 days post-immunization (d.p.i.), followed by CD4^+^ T cell enrichment using MACS. A total of 5 × 10^5^ cells were first stained with I-Ab MOG_35–55_ MHC tetramer-PE (MBL, Japan), CD3, CD4, and CD44 and then analyzed by flow cytometry.

### 2.5 EAE induction protocol

EAE was induced as previously described (21). Briefly, mice were immunized subcutaneously with 200 μg of MOG_35-55_ peptide (GenScript, CA, USA) emulsified in Complete Freund’s Adjuvant (Thermo Scientific). Pertussis toxin (200 ng; Sigma-Aldrich) was administered intraperitoneally on days 0 and 2 post-immunization. Clinical symptoms were monitored daily using the following scale: 0, no sign of clinical disease; 1, paralysis of the tail; 2, paralysis of one hindlimb; 3, paralysis of both hindlimbs; 4, paralysis of the abdomen; 5, death.

### 2.6 CD4^+^ T cells isolation, co-culture, and Th differentiation

Various subpopulations of CD4^+^ T cells from multiple organs of 2-3-month-old *Gr/fr* mice were sorted via fluorescence-activated cell sorting (FACS) using a FACSAria fusion II flow cytometer system (BD Biosciences). Flow cytometry analysis confirmed the purity of sorted cells. T cell-depleted splenocytes were prepared using anti-CD3 beads and magnetic-activated cell sorting (MACS). The sorted CD4^+^ T cells were co-cultured with the T cell-depleted splenocytes at a 1:4 ratio in the presence of soluble anti-CD3 and anti-CD28 mAbs (3 µg/mL) for 3 days. After co-culture, supernatants were collected for cytokine measurement using enzyme-linked immunosorbent assay (ELISA), while cells were stimulated with PMA, Ionomycin, and GolgiPlug for 5 h and analyzed by flow cytometry.

For Th differentiation, naïve (CD62L^+^CD44^−^) YFP^-^ CD25^−^CD4^+^ T cells from the spleens of *Gr/fr* mice were FACS sorted and differentiated into ThGM and Th1 cells, as previously described (8). Briefly, naïve T cells were cultured at a ratio of 1:4 with T cell-depleted splenocytes at a density of 1x10^6^ cells/ml. Naïve T cells were activated with soluble anti-CD3 and anti-CD28 mAbs (3 µg/mL) for 3 days in different Th differentiation conditions: ThGM: anti-IFN-γ (10 μg/ml), anti-IL-12 (10 μg/ml), anti-IL-4 (5 μg/ml) Abs, and Th1: IL-12 (20 ng/ml).

### 2.7 Bulk mRNA sequencing

YFP^+^, YFP^−^ naïve (CD62L^+^CD44^−^), and T_EM_ (CD62L^−^CD44^hi^) CD4^+^ T cells from the spleens of *Gr/fr* mice (n = 3) were FACS sorted. Then, RNA was extracted using the RNeasy Mini Kit (Qiagen) according to the manufacturer’s instructions. Libraries were prepared using 200 ng of total RNA and the Illumina TruSeq Stranded Total RNA library preparation kit. Libraries’ qualities were evaluated using the PerkinElmer Labchip GX and qPCR using the Kapa Library Quantification Kit and the Life Technologies Viia7 Real-time PCR instrument. Libraries at a concentration of 2nM were sequenced on the Illumina HiSeq2500 using the High Output v4 chemistry. Raw FASTQ sequencing reads were mapped against the mm10 reference genome using the DRAGEN genome pipeline. BAM files were used to generate a feature/gene counts matrix using the R/Bioconductor package Rsubread. Differential expression contrasts were performed using the DESeq2 package in R/Bioconductor. Differentially expressed genes (DEGs) were identified as those with Benjamini-Hochberg adjusted p ≤ 0.05 and absolute log2 fold change ≥ 2. All plots were generated using R/Bioconductor.

### 2.8 Statistical analysis

Statistical analysis was conducted using GraphPad Prism 8 software. Data was analyzed using an unpaired, two-tailed Student’s t-test between two groups and a one-way ANOVA between three or more groups. P ≤ 0.05 was considered significant.

## 3 Results

### 3.1 Varying levels of past and current GM-CSF expression in immune cells across different organs

**Figure 1:**
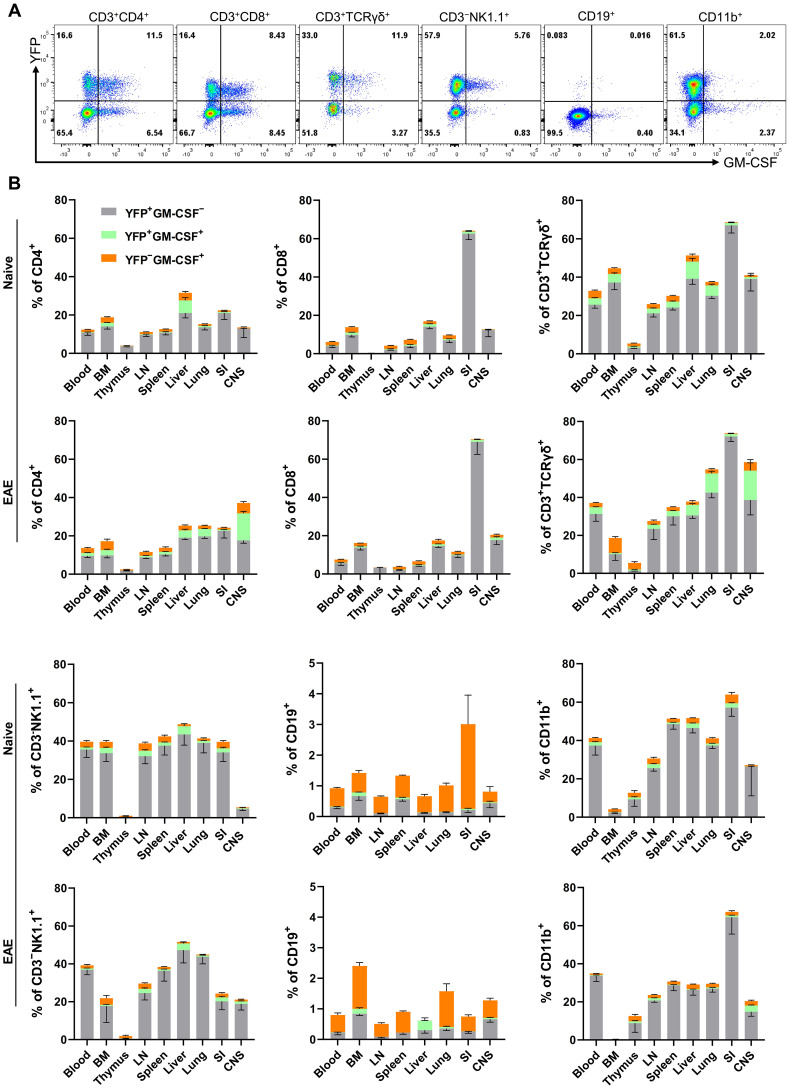
GM-CSF and YFP expression by immune cells from mouse organs. Mononuclear cells were isolated from the blood, BM, thymus, LNs, spleen, liver, lung, SI, and CNS of both naïve mice and mice with EAE at the peak of clinical disease. All samples were obtained from 2–3-month-old male and female Gr/fr mice. Cells were stimulated with PMA, Ionomycin, and GolgiPlug, and stained for CD45, lineage-specific surface markers, and GM-CSF. Cells were then analyzed by flow cytometry. YFP and GM-CSF expression were assessed within gated live CD45^hi^ populations. **(A)** Flow cytometry plots show YFP and GM-CSF expression in liver immune cells of naïve mice; a similar gating strategy was used for immune cells from other organs. **(B)** The stacked bar charts show the frequencies of YFP^+^ and GM-CSF^+^ cells among major immune cell types of naïve mice and mice with EAE at the peak of clinical disease severity. Data represent three independent experiments in naïve mice (total n = 13) and two independent experiments in mice with EAE (total n = 8). Error bars indicate the mean ± SEM. LN, lymph nodes; BM, bone marrow; SI, small intestine; CNS, central nervous system.

The numbers and phenotypic properties of GM-CSF-producing immune cells in various tissues and their fates in steady state and inflammation remain incompletely understood. To address this knowledge gap, we used our *Gr/fr* mice to investigate GM-CSF expression across different immune cell populations. Multiple immune cell types produced GM-CSF, including CD4^+^, CD8^+^, γδ T, NK, B, and CD11b^+^ cells (**Figure 1A**; general gating strategy is shown in **Supplementary Figure S2B**). Notably, there were substantial differences in past and current GM-CSF expression among immune cell types within the same anatomical location, as well as between the same cell types across different organs. For instance, while only a small portion of B cells expressed GM-CSF, up to 80% of γδ T cells expressed it. Similarly, past GM-CSF production by CD8^+^ T cells ranged from a small percentage in the LNs to 70% in the SI (**Figure 1B**).

In naïve mice, CD4^+^ T cells and NK cells in the liver exhibited higher proportions of ongoing and past GM-CSF expression than in other organs, while CD8^+^ and γδ T cells in the SI displayed elevated past GM-CSF expression. Additionally, greater proportions of CD11b^+^ cells in the SI, spleen, and liver showed GM-CSF expression than in other organs (**Figure 1B**). Notably, less than 1% of microglia expressed GM-CSF, either currently or in the past (**Supplementary Figure S2C**). In the CNS of mice with EAE at the peak of clinical disease severity, approximately 40% of CD4^+^ T cells and 60% of γδ T cells expressed GM-CSF, with ongoing expression being notably higher compared to other organs (**Figure 1B**).

**Figure 2:**
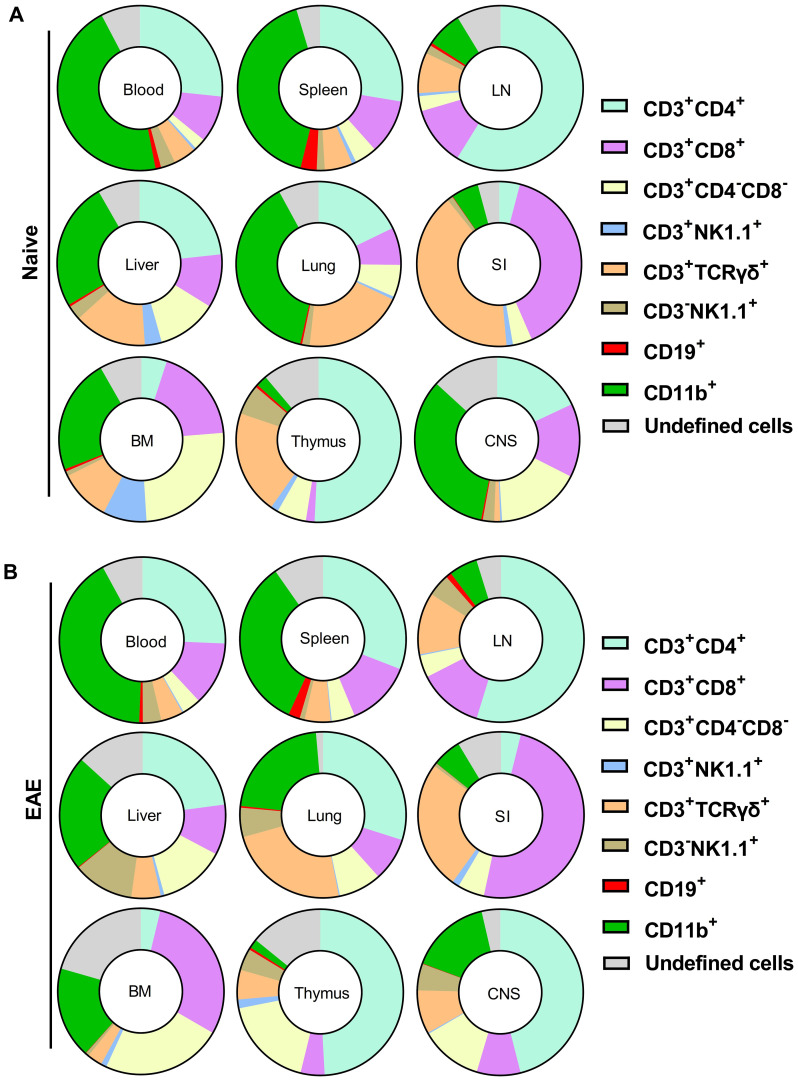
Proportions of cell types among CD45^hi^YFP^+^ immune cells of naïve **(A)** and **(B)** Gr/fr mice with EAE at the peak of clinical disease severity. Mononuclear cells were isolated from the blood, BM, thymus, LN, spleen, liver, lung, SI, and CNS of both naïve mice and mice with EAE at the peak of clinical disease. All samples were obtained from 2–3-month-old male and female Gr/fr mice. Cells were stained for CD45, lineage-specific surface markers, and analyzed by flow cytometry. Proportions of YFP^+^ cells were determined within gated live CD45^hi^ populations. The pie charts show the frequencies of major cell types among the CD45^hi^YFP^+^ immune cells. For details, see **Supplementary Table S2**. Data is presented as the mean percentage from three independent experiments in naïve (total n = 13) and two independent experiments in EAE mice (total n = 8). The “undefined” cells include rare populations such as CD3^+^CD19^+^, CD3^-^CD19^low^, CD3^+^CD8^low^, CD3^+^CD4^low^, and CD3^+^CD4^+^CD8^+^ cells. LN, lymph nodes; BM, bone marrow; SI, small intestine; CNS, central nervous system.

The primary populations with high proportions of YFP^+^ cells, indicative of prior GM-CSF production, include CD4^+^, CD8^+^, γδ T cells, and CD11b^+^ cells. Among CD45^hi^YFP^+^ cells, the dominant populations varied by organ (**Supplementary Table S2**). In the blood, spleen, and lungs of naïve mice, CD11b^+^ cells constituted the main YFP^+^ population, followed by CD4^+^ T cells. In the LNs and thymus, CD4^+^ T cells predominated (**Figure 2A**). In the liver, both CD11b^+^ and CD4^+^ T cells were the largest YFP^+^ populations, while in the SI, γδ T cells and CD8^+^ T cells were the primary YFP^+^ populations. Within the CD45^hi^YFP^+^ population, NKT and B cells represented rare subsets, while NK cells accounted for 1–17% across various organs. Additionally, a subset of YFP^+^ CD3^+^γδ^−^CD4^-^CD8^-^ cells (double negative T cells) was present in multiple organs, mostly in the BM, liver, and lung (**Figure 2**).

In EAE mice at the peak of clinical disease severity, the overall pattern of YFP expression remained similar to that of naïve mice, except in the lungs, where an increase in CD4^+^YFP^+^ cells rendered them the predominant population among YFP^+^ cells. In the CNS at the peak of EAE, CD4^+^ T cells were the most abundant YFP^+^ population (**Figure 2B**).

Among CD11b^+^ cells, most YFP^+^ cells were macrophages (CD11b^+^CD11c^+^Ly6G^−^Ly6C^−^MHC-II^−^; **Supplementary Figure S3A**) in all organs of both naïve and EAE mice. Monocytes (CD11b^+^CD11c^−^Ly6C^hi^Ly6G^−^MHC-II^−^) and cDCs (CD11b^+^CD11c^+^Ly6G^−^Ly6C^−^MHC-II^+^) constituted minor CD11b^+^YFP^+^ populations. Notably, active GM-CSF expression was restricted to cDC (**Supplementary Figure S3B**). In both naïve and mice at the peak of EAE, CD45^−^ cells did not express YFP, except for rare populations (<1%) in the lung and SI (**Supplementary Figure S3C**). In conclusion, diverse immune cells produce GM-CSF, with the primary sources being lymphoid cells, particularly CD4^+^ T cells, and macrophages among myeloid cells.

A particularly striking observation was that the vast majority of YFP^+^ cells of all types failed to re-express GM-CSF upon stimulation with PMA and Ionomycin, suggesting that GM-CSF expression is transient and possibly ceased irreversibly. Consequently, only a small subset of any immune cell type could express GM-CSF at any given time.

### 3.2 Effector memory CD4^+^ T cells are the main GM-CSF source in the lung and CNS during neuroinflammation

Given that T cells, especially CD4^+^ T cells, were the major producers of GM-CSF, we focused our studies on these cells. In the organs of both naïve and mice with EAE at the peak of clinical disease, the frequencies of CD4^+^ T cells were higher than those of CD8^+^ T cells, except in the SI, where CD8^+^ T cells predominated (**Supplementary Figures S4A, B**). In lymphoid organs, blood, and lungs of naïve mice, conventional (Foxp3^−^) CD4^+^ and CD8^+^ T cell subsets were predominantly CD62L^+^CD44^−^, indicative of a naïve phenotype. In contrast, in the SI, the frequency of naïve cells was less than 5%. The proportion of CD62L^−^CD44^+^ cells, indicative of effector memory T cells, was higher in the liver, SI, and CNS. However, in the blood and lymphoid organs, only a small percentage (approximately 1-10%) of conventional CD4^+^ or CD8^+^ T cells were CD62L^−^CD44^+^. A shift toward CD62L^−^CD44^+^ cells was observed in all organs of EAE mice at the peak of clinical disease severity, most notably in the case of CD4^+^ cells in the CNS and lung (**Supplementary Figures S4C, D**), along with an increased frequency of MOG_35-55_-specific CD4^+^CD44^+^ T cells in the lung at 8 d.p.i. (**Supplementary Figures S4E, F**). This finding aligns with other reports indicating that the lung plays a crucial role in EAE pathogenesis by serving as a priming site where effector CD4^+^ T cells, particularly Th17 cells, undergo reprogramming. This reprogramming enhances the expression of genes encoding chemokine receptors and integrins, facilitating their migration to the CNS (22, 23).

**Figure 3:**
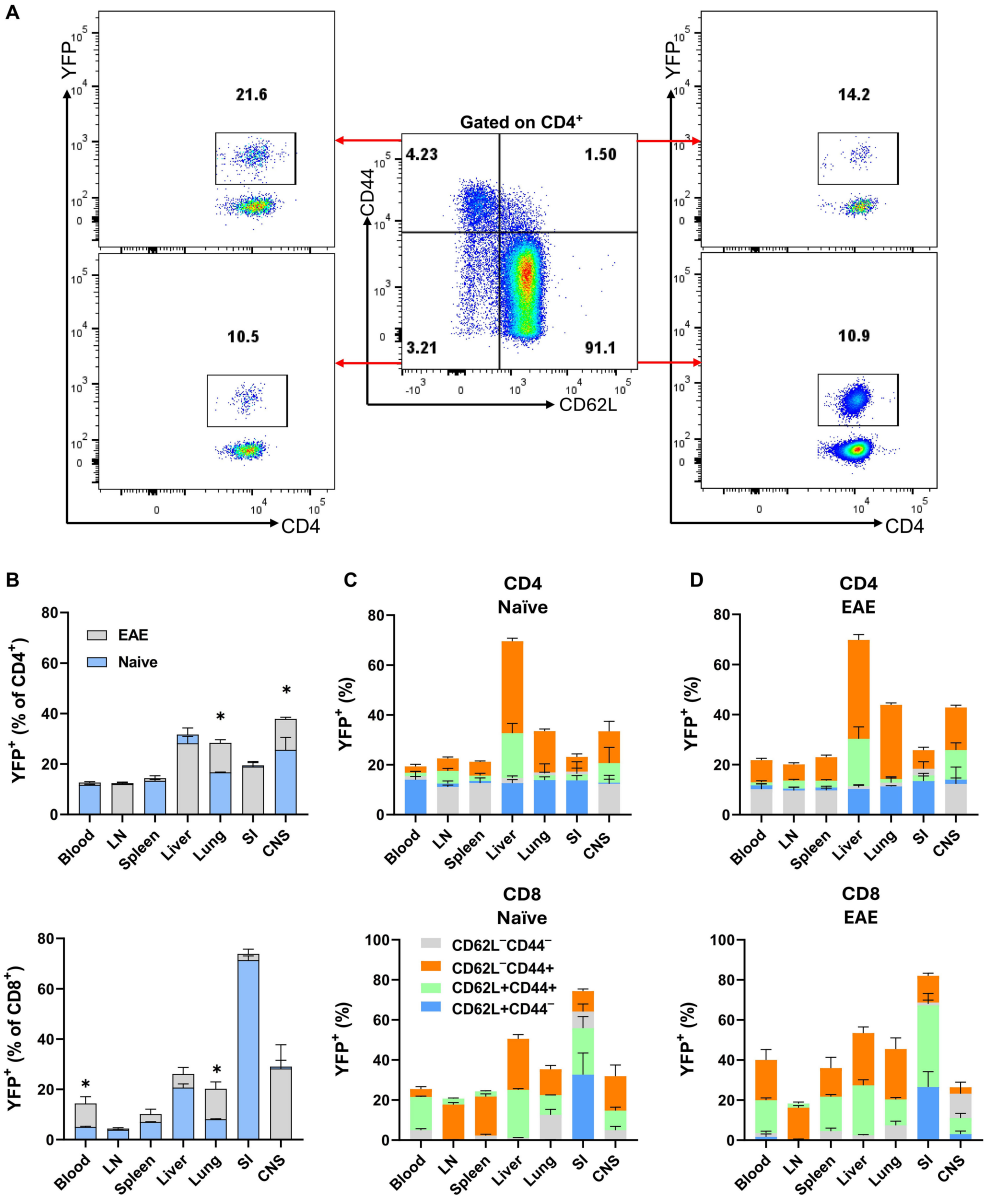
YFP expression by CD4^+^ and CD8^+^ T cells from various mouse organs. Gr/fr naïve and mice with EAE at the peak of clinical disease severity (both male and female, aged 2–3 months) were sacrificed. Cells isolated from the blood, LN, spleen, liver, lung, SI, and CNS were stained and analyzed by flow cytometry for CD45, CD4, CD8, CD62L, CD44, and YFP expression. **(A)** The gating strategy for YFP expression analysis among CD62L^−^CD44^−^, CD62L^+^CD44^−^, CD62L^−^CD44^+^, and CD62L^+^CD44^+^ subsets within conventional (Foxp3^−^) CD4^+^ T cells from the LNs of naïve mice. **(B)** Superimposed bar charts illustrate the frequencies of YFP^+^ cells in total conventional CD4^+^ and CD8^+^ T cells of naïve mice and mice with EAE. The superimposed bar charts illustrate frequencies of YFP^+^ cells in CD62L^−^CD44^−^, CD62L^+^CD44^−^, CD62L^−^CD44^+^, and CD62L^+^CD44^+^ subsets of conventional CD4^+^ (upper) and CD8^+^ (lower) T cells from **(C)** naïve mice and **(D)** mice with EAE. Data represent two independent experiments with naïve (total n = 7) and mice with EAE (total n = 8). Error bars indicate the mean ± SEM. Statistical analysis was conducted using the Student’s t-test; *P<0.05.

Using *Gr/fr* mice, we next investigated GM-CSF expression in subpopulations of conventional CD4^+^ and CD8^+^ T cells (**Figure 3A**) across various organs of both naïve and EAE-affected mice. The highest frequencies of YFP^+^CD4^+^ T cells were observed in the liver and CNS of naïve mice, with a noted increase in the lung and CNS of mice at disease peak (**Figure 3B**). The frequency of YFP^+^ cells among CD4^+^FoxP3^-^ cells in the CNS progressively increases from 8 to 27 d.p.i., suggesting a dynamic accumulation of GM-CSF-expressing T cells during disease progression, whereas only a modest increase was observed in the spleen (**Supplementary Figure S4G**). For YFP^+^CD8^+^ T cells, the highest frequency was found in the SI, with no significant changes post-immunization. Within conventional CD4^+^ and CD8^+^ T cell subsets, YFP^+^ cells were most prevalent in the effector memory (CD62L^−^CD44^+^) subsets of both naïve (**Figure 3C**) and EAE-affected mice (**Figure 3D**), with higher frequencies in the lung and CNS of mice with EAE. There was little difference in the proportions of YFP^+^CD8^+^ T cells between naïve and EAE-affected mice. In conclusion, GM-CSF-expressing effector memory CD4^+^ T cells were notably enriched in the lung and CNS of mice with EAE compared to naïve mice.

An unexpected finding was that 10-20% of naïve CD4^+^ cells (CD62L^+^CD44^-^) were YFP^+^, indicating that these cells expressed GM-CSF. This was surprising given that naïve T cells do not express cytokines, including GM-CSF. We hypothesized that these YFP^+^ naïve CD4^+^ T cells could be stem cell memory T cells (TSCM), so we stained splenocytes of naïve mice for TSCM markers, including Sca-1, and CD122, as well as other T cell subset markers such as CD95, CD27, and CD127. However, we did not find differences between YFP^+^ and YFP^-^ naïve CD4^+^ T cell populations (**Supplementary Figures S5A, B**). Additionally, no significant differences were found in current GM-CSF, IFN-γ, IL-17, or IL-2 expression between these populations following stimulation with PMA and Ionomycin (**Supplementary Figure S5C**), suggesting that YFP^+^ CD4^+^CD62^+^CD44^−^ T cells are genuinely naïve. It is unclear what led these cells to express GM-CSF, and if some naïve T cells also express other cytokines.

### 3.3 T cells simultaneously express both GM-CSF and CXCR6

**Figure 4:**
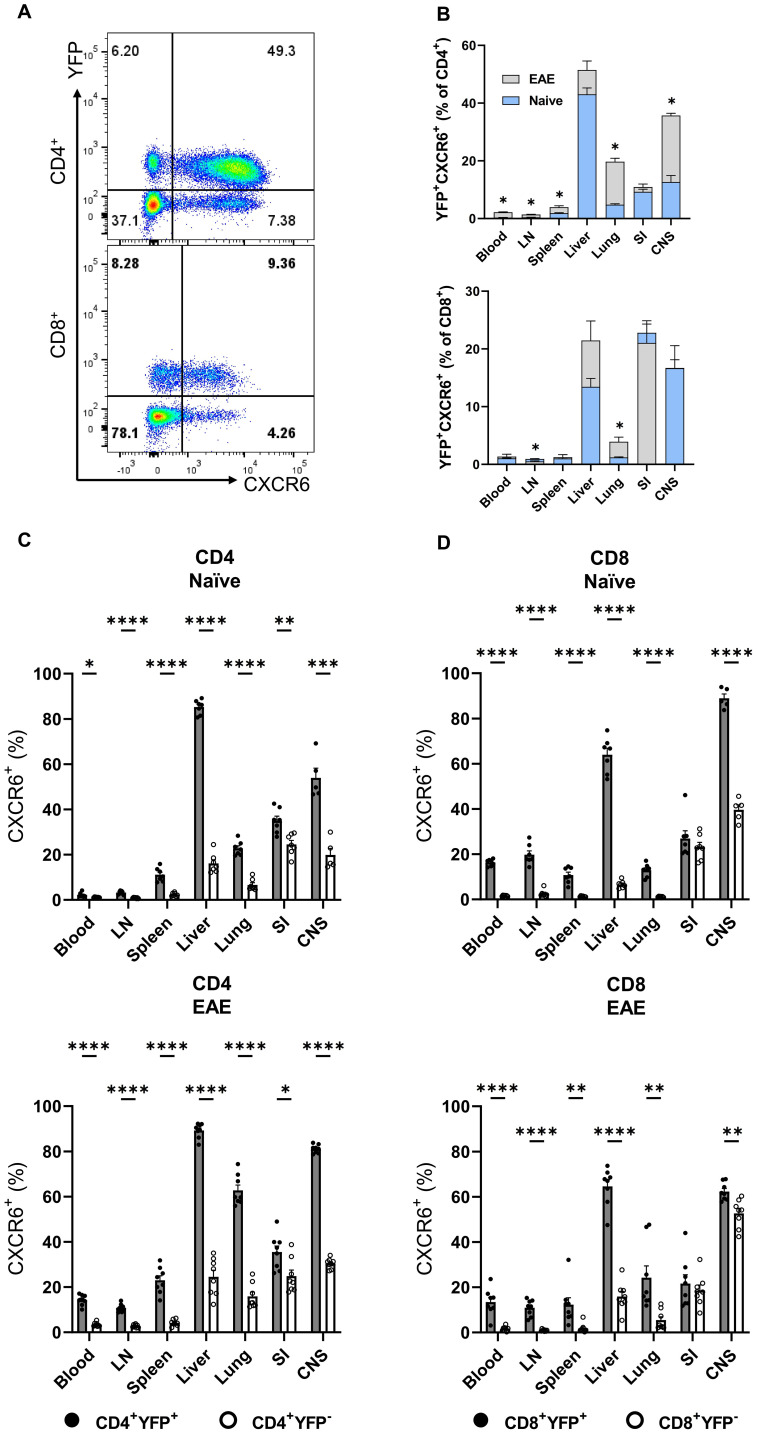
YFP and CXCR6 expression by CD4^+^ and CD8^+^ T cells. Mononuclear cells were isolated from the blood, LNs, spleen, liver, lung, SI, and CNS of 2-3-month-old male and female Gr/fr mice. The cells were stained for CD45, CD4, CD8, CD62L, CD44, and CXCR6, and evaluated for YFP expression within different subsets. **(A)**, Representative flow cytometry dot plots show YFP and CXCR6 expression by gated conventional (FoxP3^−^) CD4^+^ (upper panel) and CD8^+^ (lower panel) T cells from the liver of naïve mice. **(B)**, Superimposed bar charts for YFP^+^CXCR6^+^ cells of gated CD4^+^ (upper panel) and CD8^+^ (lower panel) T cells of naïve and mice with EAE at the peak of clinical disease severity. **(C)**, Percentages of YFP^+^CXCR6^+^ and **(D)**, YFP^−^CXCR6^−^ CD4^+^, and CD8^+^ T cells from naïve (upper panels) and mice with EAE (lower panels). Data represent two independent experiments with naïve (total n = 7) and mice with EAE (total n = 8). Error bars indicate the mean ± SEM. Statistical analysis was conducted using the Student’s t-test; *P<0.05; **P<0.01; ***P<0.001; ****P<0.0001. LN, lymph nodes; SI, small intestine; CNS, central nervous system.

CXCR6 plays a critical role in T cell trafficking and retention within tissues, particularly in the lungs, liver (24, 25), and CNS of mice with EAE (26). Recent studies have suggested a link between CXCR6 expression and GM-CSF production in T cells, especially CD4^+^ T cells, under specific inflammatory conditions or within certain tissue microenvironments (10). To investigate the correlation between GM-CSF and CXCR6 expression, we analyzed YFP and CXCR6 expression across various T cell subsets in steady-state and CNS inflammation. Conventional (Foxp3^−^) CD4^+^YFP^+^CXCR6^+^ T cell frequencies were higher in the liver compared to other organs of naïve mice, with no significant changes observed in mice with EAE at the peak of clinical disease severity. However, this population increased in the spleen, lung, and CNS of mice with EAE. CD8^+^YFP^+^CXCR6^+^ cells had the highest frequency in the SI, followed by the CNS and liver, with an increase in the lungs of mice with EAE (**Figures 4A, B**). The frequencies of CXCR6^+^ cells among YFP^+^CD4^+^ and YFP^+^CD8^+^ cells were several times higher than among their YFP^−^ counterparts, except in the case of CD8^+^ T cells from the SI. Approximately 20% of YFP^+^CD8^+^and YFP^−^CD8^+^ cells expressed CXCR6, regardless of immunization status (**Figures 4C, D**). Notably, CD4^+^ and CD8^+^ T cells from the liver and CNS exhibited the highest proportion of CXCR6 expression among YFP^+^ cells (**Figures 4B-D**).

While 10-20% of naïve (CD62L^+^CD44^−^) CD4^+^ T cells expressed YFP, this subset was vastly CXCR6^−^. In contrast, CD4^+^CD62L^+^CD44^+^YFP^+^ and CD4^+^CD62L^−^CD44^+^YFP^+^ subsets had the highest proportions of CXCR6^+^ cells across all organs in both naïve mice (**Supplementary Figure S6A**) and those with EAE at the peak of clinical disease severity (**Supplementary Figure S6B**). Our findings highlight a significant association between CXCR6 and YFP expression in T cells, except for naïve T cells. At the peak of EAE, there was almost 90% overlap between YFP and CXCR6 expression in some organs, such as the liver and CNS. We also observed similarly pronounced co-expression of YFP, CXCR3, and CXCR6 (**Supplementary Figures S6C, D**). Contradictory reports in the literature suggest that CXCR6 and CXCR3 either contribute to EAE development or have no impact (26, 27). In our study, neither CXCR3 nor CXCR6 played an important role in EAE (**Supplementary Figure S6E**).

### 3.4 A significant correlation exists between current cytokine production and the history of GM-CSF expression in T cells

**Figure 5:**
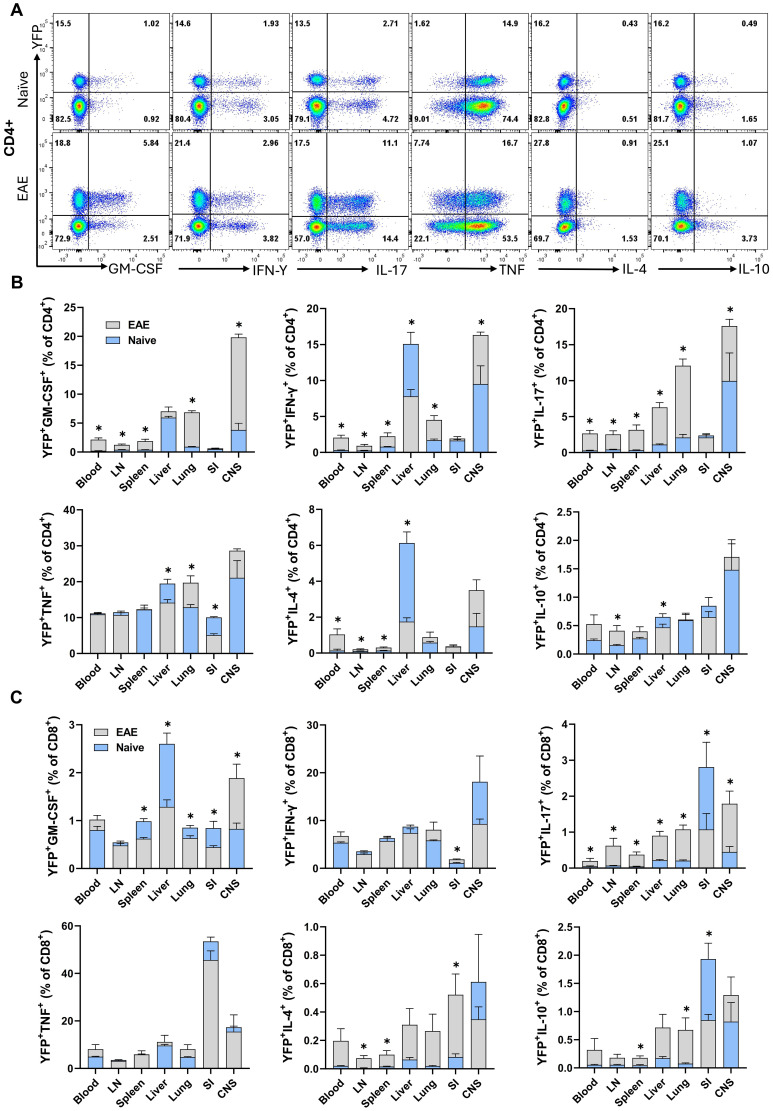
YFP and cytokine expression by CD4^+^ and CD8^+^ T cells. Mononuclear cells were isolated from the blood, LNs, spleen, liver, lung, SI, and CNS of 2–3-month-old male and female naïve and EAE Gr/fr mice. Cells were stimulated with PMA, Ionomycin, and GolgiPlug and stained for surface and intracellular antigens. **(A)**, Flow cytometry plots illustrate gating for YFP and GM-CSF, IFN-γ, IL-17, TNF, IL-4, and IL-10 in gated lung CD4^+^ T cells of naïve and mice with EAE at the peak of clinical disease severity. Superimposed bar charts illustrate YFP and cytokines GM-CSF, IFN-γ, IL-17, TNF, IL-4, and IL-10 expression by gated CD4^+^ **(B)** or CD8^+^ **(C)** T cells. Results are expressed as the mean ± SEM; naïve (total n = 7) and mice with EAE (total n = 8) from 2 independent experiments. Statistical analysis was performed using Student’s t-test; *P<0.05. LN, lymph nodes; SI, small intestine; CNS, central nervous system.

Across various organs (blood, LN, spleen, liver, lung, SI, and CNS) of both naïve and EAE mice at the peak of clinical disease severity, 10-30% of conventional CD4^+^ T cells and 10-70% of CD8^+^ T cells were YFP^+^. To assess cytokine production by YFP^+^ cells, we stimulated MNCs with PMA/Ionomycin and analyzed the expression of several cytokines by flow cytometry. Overall, except for TNF, the expressions of GM-CSF, IFN-γ, IL-17, IL-4, and IL-10 were higher in non-lymphoid organs (liver, lung, and SI) and the CNS compared to blood and lymphoid organs of both naïve and EAE mice at the peak of clinical disease severity. In general, a greater proportion of CD4^+^ T cells from mice with EAE-expressed cytokines. Following EAE induction, CD8^+^ T cells showed an upregulation in IL-17, IL-4, and IL-10 expression (**Supplementary Figure S6**). Comparison of cytokine expression between CD4^+^ YFP^+^ and YFP^−^ cells revealed a marked tendency of YFP^+^ cells from naïve and EAE mice to produce cytokines, particularly GM-CSF, IL-17, and IFN-γ (**Supplementary Figure S8**). This was not surprising, as the frequency of cells with an effector memory phenotype (CD44^+^) was higher among YFP^+^ cells (~27% in the spleen of naïve mice) compared to YFP^-^ cells (~17% in the spleen of naïve mice), with the difference being approximately two-fold higher in non-lymphoid organs. In the CD8^+^ cells of both naïve and EAE mice at the peak of clinical disease severity, a markedly bigger proportion of YFP^+^ cells produced GM-CSF and IFN-γ compared to YFP^−^ cells (**Supplementary Figure S9**). We found a significant association between YFP expression and the production of GM-CSF, IFN-γ, and IL-17 by CD4^+^ T cells from the liver, lung, and CNS of naïve mice, with a notable increase in their frequency in EAE mice (**Figures 5A, B**). Overall, CD4^+^YFP^+^ T cells constitute a multi-cytokine-producing subset primarily enriched for the expression of GM-CSF, IFN-γ, TNF, and IL-17, with minimal expression of IL-4 and IL-10. A similar increase in IL-17 expression by CD8^+^YFP^+^ cells was noted after immunization for EAE induction (**Figure 5C**).

### 3.5 GM-CSF expression is a transient characteristic of CD4^+^ T cells under steady-state conditions

**Figure 6:**
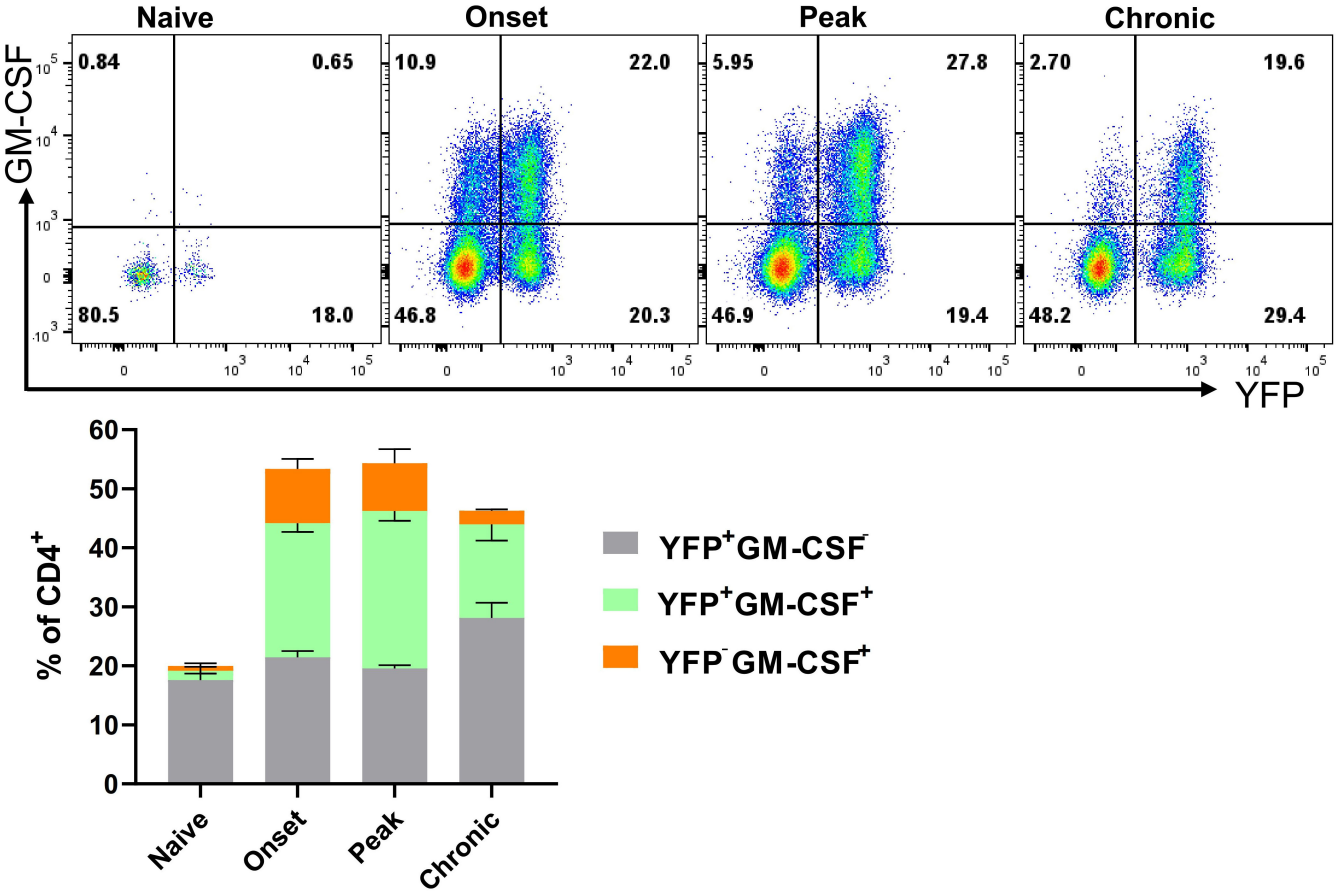
YFP and GM-CSF expression in CD4^+^ T cells from the CNS of EAE mice. Mononuclear cells were isolated from the CNSs of 2–3-month-old male and female naïve mice and mice during the onset (15 d.p.i.), peak (20 d.p.i.), and chronic (27 d.p.i.) phases of EAE. Cells were stimulated with PMA, Ionomycin, and GolgiPlug for 6 h, followed by staining for surface and intracellular antigens. Flow cytometry plots illustrate the gated YFP and GM-CSF expression within gated CD4^+^ T cells among live CD45^hi^ cells. Results for the YFP^−^GM-CSF^+^, YFP^+^GM-CSF^+^, and YFP^+^GM-CSF^−^ populations are presented as mean ± SEM by the stacked bar chart. Data represent naïve and mice with EAE (n = 4 per group) from one experiment.

Only a portion (2-18%) of CD4^+^YFP^+^ T cells from the organs of naïve mice produced GM-CSF upon ex vivo stimulation with PMA and Ionomycin, while even less YFP^-^ cells expressed GM-CSF (1-5%) (**Supplementary Figure S8A**). This indicates that GM-CSF expression is transient and possibly ceases in almost all the cells that express it. Furthermore, ThGM and Th1 cells that developed *in vitro* started to lose capacity for GM-CSF production after 72 h (**Supplementary Figure S10**). However, we hypothesized that short-term ex vivo stimulation with PMA and Ionomycin may not reflect the behavior of cells upon activation through their T cell receptor (TCR) over several days. To investigate this *in vivo* within the context of EAE, we analyzed GM-CSF and YFP expressions in the CNS at disease onset (15 d.p.i.), peak (20 d.p.i.), and beginning of chronic phase (27 d.p.i.). The frequency of GM-CSF^+^CD4^+^ T cells decreased from 32% at the onset to 18% at the chronic phase. Conversely, YFP^+^GM-CSF^−^ cells increased from 21% at the onset to 28% during the chronic phase (**Figure 6**).

**Figure 7:**
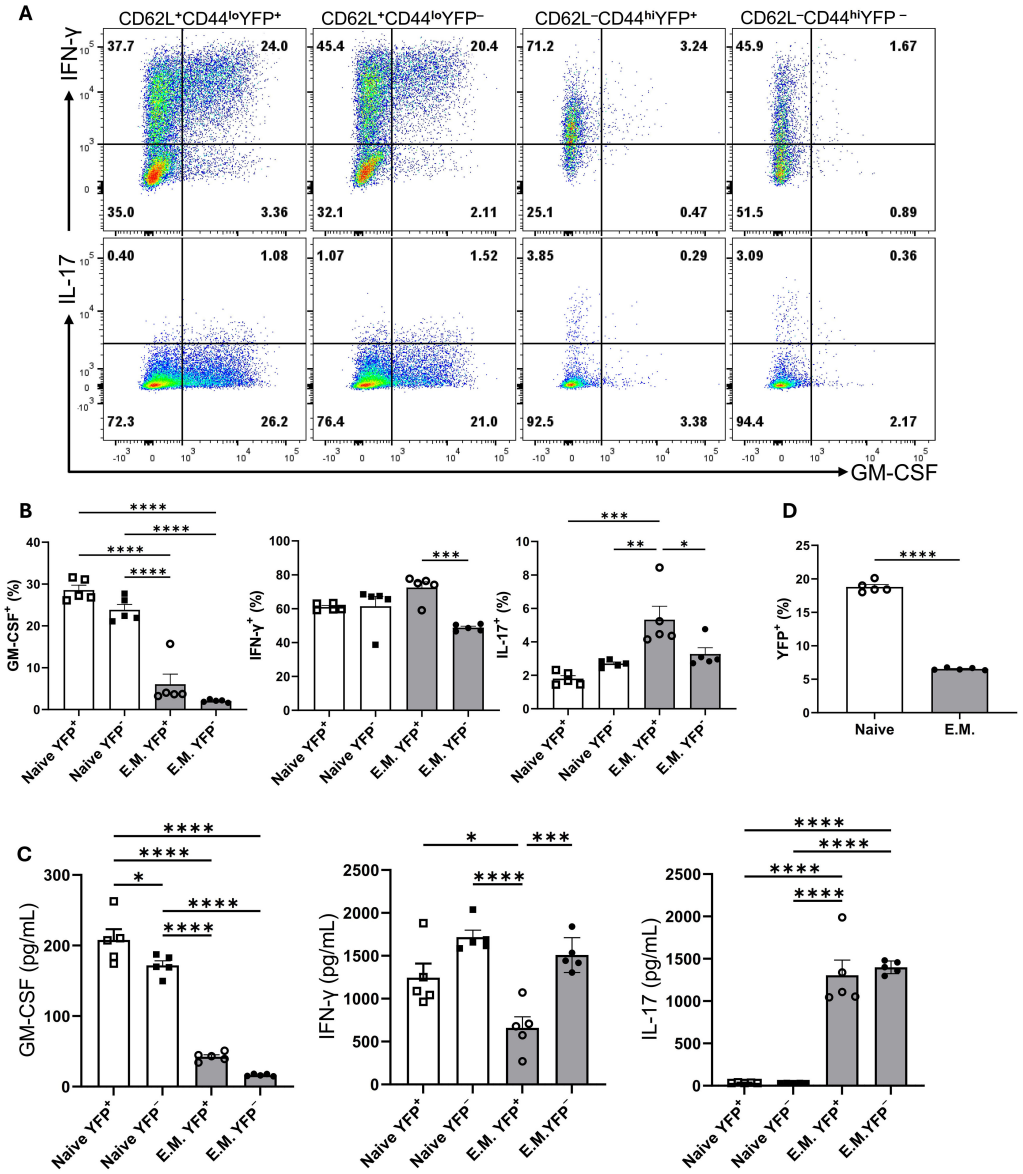
GM-CSF, IFN-γ, and IL-17 expression by YFP^+^ and YFP^−^ populations of naïve and effector memory CD4^+^ T cells following stimulation. Conventional naïve (CD25^−^CD62L^+^CD44^lo^CD4^+^) YFP^+^ and YFP^−^, and effector memory (CD25^−^CD62L^-^CD44^hi^CD4^+^) YFP^+^ and YFP^−^ T cells were sorted from the spleens of naïve 2-3-month-old male and female Gr/fr mice. The cells were co-cultured with T cell-depleted splenocytes and soluble anti-CD3/CD28 mAbs (3 µg/ml) for 72 h, then stimulated with PMA, Ionomycin, and GolgiPlug. Cells were stained against CD4, GM-CSF, IFN-γ, and IL-17. **(A)** Flow cytometry plots show gating for GM-CSF, IFN-γ, and IL-17 in gated CD4^+^ T cells. **(B)** Cytokine expression in gated CD4^+^ T cells from cultured cells following anti-CD3/CD28 mAbs stimulation. **(C)** Cytokine levels were measured by ELISA in the culture supernatants after anti-CD3/CD28 mAbs stimulation. **(D)** The proportions of YFP^+^ cells in naïve and effector memory YFP^−^ populations after 72 h of activation and co-culture. Data are presented as the mean ± SEM from 2 independent experiments (total n=5). Statistical analysis was conducted using an unpaired Student’s t-test and one-way ANOVA. *P < 0.05; **P < 0.01; ***P < 0.001; ****P < 0.0001. E.M., effector memory CD4^+^ T cell.

To test if YFP^+^CD4^+^T cells restart their GM-CSF expression upon activation through their TCR, we sorted YFP^+^ and YFP^−^ naïve and effector/memory CD4^+^ T cells from the spleen of naïve mice. Following 3 days activation with anti-CD3/28 mAbs, flow cytometry analysis revealed both YFP^+^ and YFP^−^ naïve CD4^+^ T cells similarly expressed GM-CSF (25-30%), whereas both YFP^+^ and YFP^−^ effector memory CD4^+^ T cells barely expressed GM-CSF (2-5%) (**Figures 7A, B**). GM-CSF concentrations in culture supernatants agreed with flow cytometry data (**Figure 7C**). The percentages of IFN-γ^+^ and IL-17^+^ cells were similar among all four tested populations. Interestingly, both YFP^+^ and YFP^−^ subsets of effector memory CD4^+^ T cells secreted notable amounts of IL-17 in culture supernatants, while naïve cells secreted almost none (**Figure 7C**). In addition, the rate of naïve CD4^+^YFP^−^ T cells converting to YFP^+^ was greater than that of effector memory YFP^−^CD4^+^ T cells (**Figure 7D**). It is surprising that effector memory cells, even those that previously expressed GM-CSF, did not re-express it upon activation. However, we noticed that effector memory cells in our cultures rapidly declined after activation and that only a fraction survived for three days. Hence, it is possible that effector memory cells that produced GM-CSF had already died by the time of analysis and are therefore not represented in our data. However, when splenocytes of naïve mice are directly ex vivo stimulated with PMA and Ionomycin, there are almost no GM-CSF^+^ cells among YFP^+^CD4 T cells (**Figure 5B**), suggesting that effector memory YFP^+^CD4^+^ T cells indeed do not express GM-CSF. While GM-CSF expression appears transient and is not readily re-inducible ex vivo under conditions we had tested, this does not exclude the possibility of re-induction *in vivo* or under different conditions.

### 3.6 The transcriptomes of YFP^+^ and YFP^−^ naïve CD4^+^ T cells are highly similar, whereas those of YFP^+^ and YFP^−^ CD4^+^ effector memory T cells are markedly distinct

**Figure 8:**
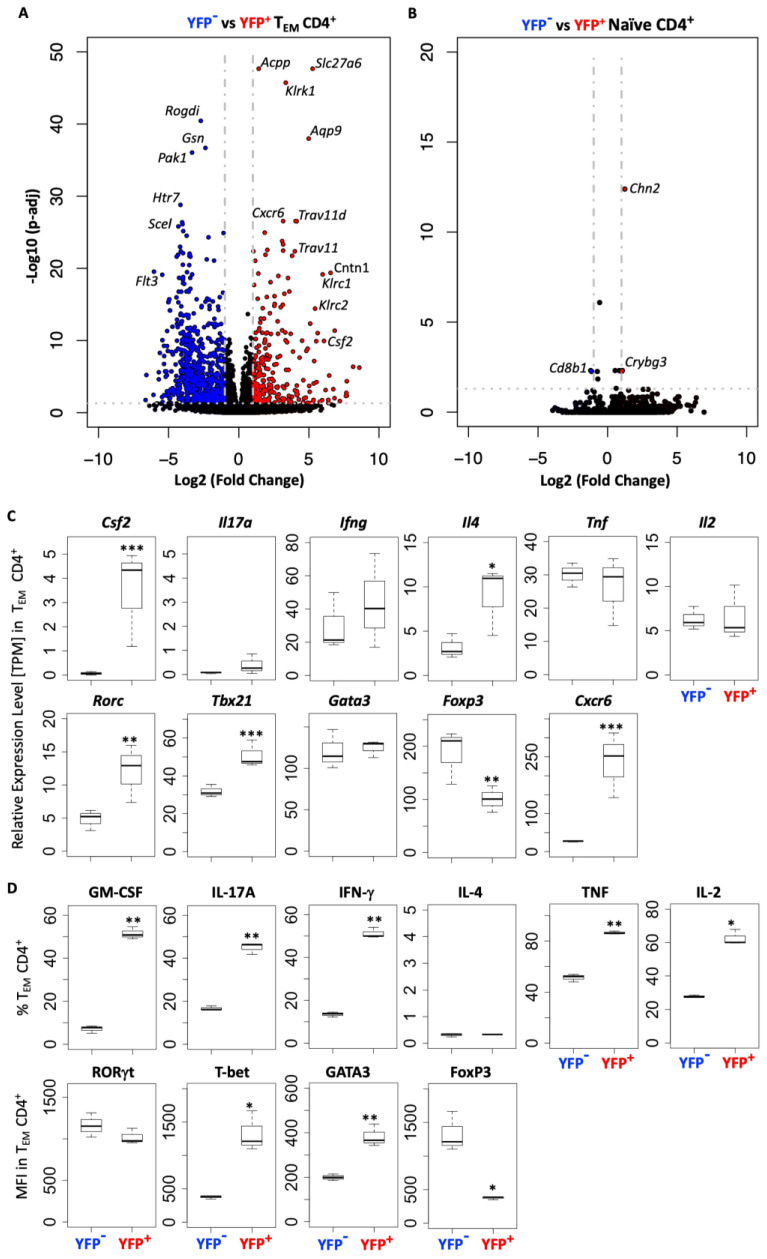
YFP^+^ and YFP^-^ CD4^+^ T_EM_ cells have a distinct transcriptomic profile. YFP^−^ and YFP^+^ naïve (CD62L^+^CD44^lo^) and T_EM_ (CD62L^−^CD44^hi^) CD4^+^ T cells were FACS sorted from the spleens of naïve *Gr/fr* mice (n=3), RNA was isolated and analyzed by RNA-seq. **(A)** Volcano plot shows 914 differentially expressed genes between YFP^+^ and YFP^−^ CD4^+^ T_EM_ cells, with 262 genes upregulated in YFP^+^ and 652 genes in YFP^−^ cells. **(B)** Volcano plot shows 3 differentially expressed genes between YFP^+^ and YFP^−^ naïve CD4^+^ T cells, with 2 genes upregulated in YFP^+^ and 1 gene in YFP^−^ cells. **(C)** Normalized gene expression levels (TPM) of several markers expressed by YFP^+^ and YFP^−^ CD4^+^ T_EM_ cells from RNA-seq analysis. **(D)** YFP^+^ and YFP^−^ CD4^+^ T_EM_ cells from the CNS of *Gr/fr* mice (n=3) immunized for EAE induction were analyzed at the peak of clinical disease severity. Box plots show frequencies of GM-CSF^+^, IL-17A^+^, IFN-γ^+^, IL-4^+^, TNF^+^, and IL-2^+^ YFP^+^ and YFP^−^ CD4^+^ T_EM_ cells in the CNS of mice with EAE. The bottom row box plot shows the staining intensity (MFI) of RORγt, T-bet, GATA3, and Foxp3 in YFP^+^ and YFP^−^ CD4^+^ T_EM_ cells in the CNS of mice with EAE. Box plots show interquartile range (IQR), with horizontal lines denoting the median. P-values were calculated using paired Student’s t-test; *p < 0.05, **p < 0.01, ***p < 0.001. T_EM_, effector memory CD4^+^ T cell; MFI, mean fluorescence intensity.

To identify differences between CD4^+^ T cells that did or did not produce GM-CSF^+^, we sorted YFP^+^ and YFP^−^ naïve and effector memory CD4^+^ T cells from the spleens of naïve *Gr/fr* mice and analyzed their transcriptomes using bulk RNA-seq. Comparison between CD4^+^ effector memory subpopulations identified 262 genes differentially upregulated in YFP^+^ and 652 genes in YFP^−^ cells (**Figure 8A**), while only a few genes were differentially expressed between YFP^+^ and YFP^−^ naïve CD4^+^ T cells (**Figure 8B**). YFP^+^ CD4^+^ effector memory T cells showed higher expression of *Csf2*, *Il4*, *Rorc*, *Tbx21*, and *Cxcr6* with lower expression of *Foxp3* (**Figures 8A, C**). On the other hand, YFP^+^ naïve CD4^+^ T cells only showed higher expression of *Chn2* and *Crybg3* and lower expression of *Cd8b1* compared to YFP^+^ naïve CD4^+^ T cells (**Figure 8B**). We next tested some of these findings from the steady state in mice with EAE at the peak of clinical disease severity. In agreement with RNA-seq results, most YFP^+^ CD4^+^ effector memory T cells from the CNS of mice with EAE expressed GM-CSF. They also displayed higher expression of *T-bet* and lower expression of *Foxp3* (**Figure 8D**). Unlike YFP^+^ CD4^+^ effector memory T cells in a steady state, these EAE cells had higher frequencies of IL-17^+^, IFN-γ^+^, TNF^+^, and IL-2^+^ cells and higher expression of *GATA3* (**Figure 8D**). Collectively, these results show that YFP^+^ and YFP^-^ naïve CD4^+^ T cells are virtually indistinguishable, whereas YFP^+^ and YFP^-^ CD4^+^ effector memory T cells differ to a surprisingly large extent, both in a steady state and in inflammation.

## 4 Discussion

GM-CSF is a key pro-inflammatory cytokine that elicits the inflammatory phenotype in myeloid cells. GM-CSF is produced at relatively low levels in a steady state, but its production significantly increases during immune responses. Various immune cells produce GM-CSF, with T cells being its primary source during inflammation (10, 11, 18, 20). Given its essential role in myeloid cell function during inflammation, including autoimmunity, the biology of GM-CSF has been studied extensively and from various angles. However, the relationship between past and ongoing GM-CSF expression by diverse immune cells across different organs is only partially understood. In this study, we took advantage of our GM-CSF fate mapping mouse line to trace GM-CSF-expressing cells in steady-state and autoimmune neuroinflammation. Overall, we found that the capacity to express GM-CSF is transient, as the vast majority of cells that previously expressed it do not express it again, at least not upon stimulation ex vivo. Unexpectedly, myeloid cells, primarily macrophages, were the dominant GM-CSF-producing cell type in some organs, such as the liver, lung, and spleen. In other organs, such as LNs, SI, and inflamed CNS, T cells were the leading producers of GM-CSF. Among CD4^+^ T cells, most GM-CSF-producing cells were found in the effector memory subset, as expected. Surprisingly, a subset of naïve CD4^+^ T cells also exhibited a history of GM-CSF expression. Prior GM-CSF expression by effector memory CD4^+^ T cells strongly correlated with a distinct transcriptomic profile. We also found that GM-CSF-producing T cells co-express CXCR6, suggesting a link to tissue residency.

In both naïve and EAE mice at the peak of clinical disease severity, CD4^+^ T cells, γδ T cells, NK cells, and CD11b^+^ cells had the highest proportions of GM-CSF-expressing cells (primarily past GM-CSF expression) in different organs. In contrast, B cells were unique in predominantly expressing GM-CSF actively, albeit in very low numbers. Consistent with our findings, Komuczki et al. reported that ongoing GM-CSF expression is nearly undetectable before EAE induction but emerges in LNs at disease onset, primarily in CD4^+^ and CD8^+^ T cells, γδ T cells, and NK cells. Notably, most of these cells had a history of GM-CSF expression, but only small subsets actively produced it (10). Among CD45^hi^YFP^+^ cells, the dominant populations varied by tissue. In the blood, spleen, and lungs of naïve mice, CD11b^+^ cells were the predominant YFP^+^ population, followed by CD4^+^ T cells. In LNs and the thymus, CD4^+^ T cells were the major YFP^+^ cells, whereas in the liver, both CD11b^+^ and CD4^+^ T cells were prominent. In the SI, γδ T cells and CD8^+^ T cells were the primary YFP^+^ populations. Although the distribution of GM-CSF-expressing cells largely reflects the overall frequency of immune cell subsets across tissues, our findings suggest that CD4^+^ T cells and macrophages are the predominant sources of GM-CSF. Consistent with findings from Komuczki et al. (10) and Sheng et al. (28), our results show that in the inflamed CNS, effector CD4^+^ T cells are the dominant GM-CSF producers, with smaller contributions by CD8^+^ T cells, NK cells, γδ T cells, and CD11b^+^ cells. In contrast, in inflamed joints during inflammatory arthritis, NK cells and macrophages are the primary GM-CSF producers (20, 29), while patients with spondyloarthritis exhibit increased GM-CSF-producing CD4^+^ and CD8^+^ lymphocytes in their joints, along with an expansion of GM-CSF-producing ILC3 in synovial tissues (30). ILC3 also plays a key role in GM-CSF production during intestinal inflammation (18). These findings highlight the complex interplay between immune cell type, organ-specific microenvironments, and inflammatory conditions in shaping GM-CSF expression.

In our study, 10-20% of naïve CD4^+^ T cells (but not naïve CD8^+^ T cells) were YFP^+^, indicating prior GM-CSF expression. This was unexpected, as naïve T cells typically do not produce GM-CSF (12, 31). Neither YFP^−^ nor YFP^+^ naïve CD4^+^ T cells from the spleen of naïve mice produced GM-CSF after stimulation with PMA/Ionomycin, which was in contrast to effector memory cells. Naïve CD4^+^ T cells also did not produce IFN-γ or IL-2, whereas notable numbers of effector memory cells produced them. This suggests that YFP^+^ naïve CD4^+^ T cells are indeed naïve. Further, we sorted naïve YFP^−^ and YFP^+^ CD4^+^ T cells and activated them with anti-CD3 and anti-CD28 mAbs for several days. Both subpopulations behaved similarly about the production of various cytokines, including GM-CSF, whereas they were notably dissimilar to effector memory cells. One possible explanation is that these YFP^+^ naïve CD4^+^ T cells are TSCM cells; however, we found no significant differences in TSCM markers (32) expression between YFP^+^ and YFP^-^ naïve CD4^+^ T cells. To further investigate, we compared these two cell subsets by RNA-seq. While numerous genes were differentially expressed (>900) between YFP^+^ and YFP^-^ effector memory CD4^+^ T cells, YFP^+^ and YFP^-^ naïve CD4^+^ T cells differed in the expression of only several genes. Specifically, YFP^+^ naïve CD4^+^ T cells exhibited higher expression of *Chn2* and *Crybg3* and lower expression of *Cd8b1* compared to their YFP^-^ counterparts. However, no well-established correlation exists between these genes and GM-CSF expression in the literature. Overall, our findings suggest that YFP^+^ CD4^+^CD62L^+^CD44^-^ T cells are genuinely naïve. The mechanisms whereby they expressed GM-CSF at some point remain unclear, but it appears that these cells did not undergo widespread and permanent changes in their phenotype. Since we never found YFP^+^ naïve CD8^+^ T cells, it seems unlikely that YFP expression in some naïve CD4^+^ T cells is an artifact of a “leaky” transgenic system. Future studies are needed to determine whether this subset of naïve CD4^+^ T cells has distinct biological relevance. It would be important to determine whether these cells also expressed other effector cytokines (e.g., IFN-γ) or GM-CSF expression is unique in this context. Additional investigations, including temporal analysis to determine when YFP^+^ naïve cells expressed GM-CSF, TCR repertoire analysis to identify clonal expansion or distinct TCR usage, and epigenetic profiling to evaluate whether these cells retain signatures of prior activation, may uncover novel insights into the plasticity of naïve T cells. Additionally, single-cell RNA-seq could provide a more granular resolution of subpopulations within both naïve and effector CD4^+^YFP^+^ pools.

Previous studies have suggested a link between CXCR6 expression and GM-CSF production in CD4^+^ T cells in neuroinflammation (10, 26). In this study, considerably more CD4^+^YFP^+^ T cells were CXCR6^+^ than their YFP^-^ counterparts. At EAE peak, the CXCR6^+^ populations increased in the spleen, lung, and CNS, suggesting a correlation between CXCR6 and GM-CSF expression, as well as T-cell trafficking and retention in inflamed tissues (24–26). Komuczki et al. reported that nearly half of CNS-infiltrating CD44^+^ Th cells were ex-GM-CSF^+^, with most co-expressing TNF, IFN-γ, and IL-17. GM-CSF^+^ Th cells were predominantly CXCR6^+^ and belonged to a multi-cytokine-producing subset characterized by TNF and IFN-γ co-expression (10). Other studies indicate that CD4^+^CXCR6^+^ T cells represent terminally differentiated effectors, serving as key cytokine producers in inflamed tissues. These cells proliferate rapidly and express high levels of cytolytic granule proteins (33, 34). Interestingly, nearly all CD4^+^ T cells that produced two or more cytokines, such as IL-17, GM-CSF, or IFN-γ, were CXCR6^+^. Additionally, half of IL-17 single-producers and a third of GM-CSF single-producers in the LNs of EAE mice were CXCR6^+^ (33). Consistent with previous reports, our comparison of cytokine expression between YFP^+^ and YFP^-^ CD4^+^ T cells revealed that YFP^+^ cells, both in naïve and EAE mice, exhibited a greater propensity to produce GM-CSF, IL-17, and IFN-γ and express CXCR6. Collectively, our findings confirm that YFP^+^ CD4^+^ T cells constitute a multi-cytokine-producing subset enriched for high CXCR6 expression and production of GM-CSF, IFN-γ, TNF, and IL-17, with minimal IL-4 and IL-10 expression.

Although effector CD4^+^ T cells could produce GM-CSF following activation, this was not sustained in most effector cells. CD4^+^ T cells within the CNS of EAE mice expressed GM-CSF, but as the disease progressed into the chronic phase, CD4^+^ T cells gradually lost their ability to produce GM-CSF. Consistent with this, GM-CSF production of *in vitro*-generated ThGM cells also declined after 72 h post-activation. Komuczki et al. also reported that CNS-invading CD4^+^ T cells initially expressed GM-CSF, but expression declined 14 d.p.i (10). They reported that nearly half of CNS-infiltrating effector CD4^+^ T cells were ex-GM-CSF^+^, while just 8% of effector CD4^+^ T cells exhibited ongoing GM-CSF expression, indicating a gradual downregulation of GM-CSF expression (10). Furthermore, our follow-up analysis at 27 d.p.i., corresponding to the early chronic phase, showed further accumulation of YFP^+^GM-CSF^-^ CD4^+^ T cells. These results suggest that GM-CSF expression is transient, with only a subset of CD4^+^ T cells maintaining the ability to produce GM-CSF at any given time. This limited expression likely serves as a protective mechanism to prevent excessive and potentially harmful inflammation.

In conclusion, this study revealed a complex and dynamic landscape of GM-CSF expression by primarily immune cells. Notably, while myeloid cells, particularly macrophages, and T cells were major GM-CSF producers, their relative contributions varied substantially between organs. The ability of individual cells to produce GM-CSF was largely transient, as the vast majority of cells that previously produced GM-CSF failed to produce it again upon activation. Surprisingly, effector memory Th cells that produced GM-CSF had a dramatically different transcriptome from those that never produced GM-CSF. This indicates that the factors that drive GM-CSF expression also give rise to numerous other phenotypic traits. The history of GM-CSF expression in some naïve CD4^+^ T cells is a novel and intriguing observation that warrants further investigation. Finally, we confirmed a strong association between CXCR6 expression and GM-CSF production in CD4^+^ T cells, linking this marker to a multi-cytokine-producing effector subset enriched in inflamed tissues. Future studies should focus on elucidating the molecular mechanisms governing the transient nature of GM-CSF expression, as well as the origin and relevance of GM-CSF expression in naïve CD4^+^ T cells.

## Data Availability

RNA-seq data from Gr/fr mice have been deposited in the NCBI Gene Expression Omnibus (GEO) database under accession number GSE305413.
