# Supplementary material for: GM-CSF production by immune cells in steady state and autoimmune neuroinflammation mapped using fate reporting mice
Source: Front Immunol. 2025 Aug 19;16:1617074. doi: 10.3389/fimmu.2025.1617074 (PMC12401695; doi:10.3389/fimmu.2025.1617074)
Supplement: Supplementary file 1 [file DataSheet1.docx]

**Supplemental material**

**Fig. S1. Schematic representation of the GM-CSF fate-mapping system.** Gr mice were developed by replacing the wild-type (WT) *Csf2* gene with a transgene that enables fate mapping of GM-CSF-expressing cells. (**A**) Schematic representation of the WT *Csf2* gene, consisting of four exons. (**B**) Structure of the *Csf2* locus after targeted replacement with a transgene containing an IRES-iCre-2A-BFP-pA cassette inserted between exon 4 and 3′UTR, allowing expression of iCre recombinase and BFP under the control of the endogenous *Csf2* promoter. (**C**) Gr mice were crossed with Rosa26-eYFP reporter mice, in which a loxP-flanked STOP cassette prevents YFP expression. (**D**) Upon GM-CSF expression, iCre is expressed, leading to the excision of the STOP cassette and the permanent expression of YFP in GM-CSF-expressing cells and their progeny. iCre: improved Cre recombinase, BFP: blue fluorescent protein, YFP: yellow fluorescent protein. This figure was created with BioRender.com.

**Figure S2. Flow cytometric analysis and gating strategy for immune cell subsets and GM-CSF expression in *Gr/fr* mice. (A)** Correlation between BFP and GM-CSF expression in CD4^+^ T cells from the CNS of mice at the peak of clinical disease severity. Mononuclear cells were obtained from the CNS of *Gr/fr* mice at the peak of EAE. The cells were stimulated for 6 h with PMA, Ionomycin, and GolgiPlug, followed by staining for surface and intracellular antigens. **(B)** Gating strategy for flow cytometric analysis of lymphoid and myeloid cells among splenocytes of *Gr/fr* mice. A similar gating strategy was used in analyses of immune cells from other organs. **(C)** Representative flow cytometry dot plot and a stacked bar chart show YFP and GM-CSF expression by gated microglial cells (CD45^low^CD11b^low^) of mice with EAE at the peak of clinical disease severity (two independent experiments, total n = 8). MΦ: macrophage, cDC: conventional dendritic cell.

**Figure S3. GM-CSF and YFP expression in CD11b^+^ subsets from mouse organs.** Mononuclear cells were isolated from the blood, BM, thymus, LNs, spleen, liver, lung, SI, and CNS of both naïve mice and mice with EAE at peak clinical disease severity. The mice used were 2-3 months old males and females. Cells were stimulated with PMA, Ionomycin, and GolgiPlug, stained for CD45, myeloid-specific surface markers, and GM-CSF, and analyzed by flow cytometry. YFP and GM-CSF expressions were quantified within gated live CD45^hi^ populations. **(A)** Proportions of major myeloid cell types among CD45^hi^CD11b^+^YFP^+^ immune cells in naïve and EAE mice. Frequencies of YFP^+^ cells were determined within gated live CD45^hi^CD11b^+^ populations. Stacked bar charts display the proportions of major myeloid cell types among these YFP^+^ cells. **(B)** The frequencies of YFP^+^ and GM-CSF^+^ cells among major myeloid cell types in naïve mice and mice with EAE. **(C)** An example of YFP expression in CD45^+^ and CD45^-^ cells among live cells (from the lung of EAE mice). Data are presented as the mean ± SEM from two independent experiments in naïve (total n = 13) and EAE (total n = 8) mice. Abbreviations: LN - lymph nodes, BM - bone marrow, SI - small intestine, CNS - central nervous system, cDC- conventional dendritic cells.

**Figure S4. CD4^+^ and CD8^+^** **T cell subset frequencies in mouse organs.** Both naïve and mice with EAE at the peak of clinical disease severity (male and female *Gr/fr* mice aged 2-3 months) were sacrificed. Their cells were isolated from the blood, LNs, spleen, liver, lung, SI, and CNS, stained, and analyzed by flow cytometry for CD45, CD4, CD8, CD62L, and CD44 expression. **(A)** An example of the gating strategy for CD4^+^CD8^−^ and CD4^−^CD8^+^ cells within live CD45^hi^ cells (from the SI of naïve mice). **(B)** Superimposed bar charts illustrate the frequencies of CD4^+^ and CD8^+^ T cells across multiple organs in naïve and EAE mice at the peak of clinical disease severity. **(C)** Examples of gating strategy for CD62L^−^CD44^−^, CD62L^+^CD44^−^, CD62L^−^CD44^+^, and CD62L^+^CD44^+^ subsets within conventional (Foxp3^−^) CD4^+^ T cells (upper panel) and CD8^+^ T cells (lower panel) from the spleen of naïve mice. **(D)** Stacked bar charts show frequencies of CD62L^−^CD44^−^, CD62L^+^CD44^−^, CD62L^−^CD44^+^, and CD62L^+^CD44^+^ subsets within total conventional (Foxp3^−^) CD4^+^ and CD8^+^ T cell populations from naïve and mice with EAE at the peak of clinical disease severity. **(E)** Cells from the spleen, liver, and lung of MOG₃₅₋₅₅-immunized mice were harvested at 8 d.p.i. (preclinical stage), stained with I-Aᵇ MOG₃₅₋₅₅ MHC tetramer for CD3, CD4, and CD44 markers before analysis by flow cytometry. Representative dot plots for the spleen, liver, and lung illustrate the percentage of tetramer⁺ cells within the CD4⁺CD44⁺ T cell population. **(F)** The frequencies of MOG₃₅₋₅₅-specific CD4⁺CD44⁺ T cells among total CD4⁺CD44⁺ T cells are presented as mean ± SEM (n = 3 per group per experiment), based on data from two independent experiments. **(G)** Representative plots of CD4⁺ Foxp3^–^YFP⁺ T cells at various time points during EAE are shown (n=3-4). Data represent two independent experiments with naïve (total n = 7) and EAE (total n = 8) mice for panels A–D. Statistical analysis was performed using Student's t-test; *P<0.05; **P<0.01; ***P<0.001. Abbreviations: LN- lymph nodes, SI- small intestine, CNS- central nervous system, d.p.i.- days post immunization.

**Figure S5. Comparison of naïve (CD62L⁺CD44**^−^**) YFP⁺ and YFP**^−^ **CD4⁺ T cells.** CD4⁺ T cells were isolated from the spleens of naïve 2-3-month-old male and female *Gr/fr* mice using magnetic-activated cell sorting (MACS). Cells were stained for CD4, CD62L, CD44, CD122, Sca-1, CD95, CD27, and CD127, and analyzed by flow cytometry. **(A)** The gating strategy for Sca-1⁺CD122⁺ cells in gated live naïve (CD62L⁺CD44^−^) YFP⁺ (left) and YFP^−^ (right) CD4⁺ T cells, along with their corresponding bar graph showing summarized data from multiple mice. **(B)** Expression of CD127, CD27, and CD95 in naïve (CD62L⁺CD44^−^) YFP⁺ and YFP^−^ CD4⁺ T cells. Effector memory (CD62L^−^CD44^+^) CD4⁺ T cells were included for comparison. **(C)** The cytokine expression by YFP⁺ and YFP− populations of naïve and effector memory CD4⁺ T cells in the spleens of naïve 2-3-month-old female *Gr/fr* mice. Cells were stimulated with PMA, Ionomycin, and GolgiPlug for 5 h, followed by staining for surface and intracellular antigens. Data are presented as mean ± SEM from two independent experiments for A and B (total n = 8) and a single experiment for C (n = 3). Statistical analysis was conducted using an unpaired Student's t-test and one-way ANOVA. ****P<0.0001. Abbreviations: E.M.- effector memory CD4 T-cell.

**Figure S6. CXCR6 and YFP expression in CD4^+^ T cell subsets**. Mononuclear cells were isolated from the blood, LNs, spleen, liver, lung, SI, and CNS of 2–3-month-old male and female *Gr/fr* mice. Cells were stained for CD45, CD4, CD62L, CD44, Foxp3 and CXCR6. Conventional (Foxp3^−^) CD4⁺ T cell subsets were defined based on CD62L and CD44 expression. YFP⁺ cells were analyzed for CXCR6 expression in **(A)** naïve and **(B**) EAE mice at the peak of clinical disease severity, shown in superimposed bar charts. **(C, D)** Mononuclear cells were isolated from the CNS of mice with EAE (15 d.p.i.) and stained for surface markers. Flow cytometry plots show YFP, BFP, CXCR6, and CXCR3 expression within T_EM_ (CD62L^−^CD44^hi^) CD4⁺ cells. **(E)** Clinical scores of *Cxcr6*⁻/⁻, *Cxcr3*⁻/⁻, and wild type (WT) mice immunized for EAE induction. The data represent two independent experiments, with naïve (total n = 7) and EAE-induced (total n = 8 for panel B and total n = 10 for panel E) mice, and n = 4 from one experiment for C and D. Error bars indicate mean ± SEM. P.I. - post immunization.

**Figure S7. The cytokine expression by CD4^+^ and CD8^+^ T cells from different organs.** Mononuclear cells were isolated from the blood, LN, spleen, liver, lung, SI, and CNS of 2–3-month-old male and female *Gr/fr* mice (naïve and with EAE at the peak of clinical disease). Cells were stimulated with PMA, Ionomycin, and GolgiPlug, and stained for the surface and intracellular antigens of **(A)** CD4^+^ and **(B)** CD8^+^ T cells, shown in superimposed bar charts. Results are expressed as the mean ± SEM with naïve (total n = 7) and EAE-immunized (total n = 8) from two independent experiments. Statistical analysis was performed using Student’s t-test; *P<0.05.

**Figure S8. The cytokine expression by YFP^+^ and YFP**^−^ **CD4^+^ T cells.** Mononuclear cells were isolated from the blood, LNs, spleen, liver, lung, SI, and CNS of 2–3-month-old male and female naïve and EAE *Gr/fr* mice at the peak of clinical disease. Cells were stimulated with PMA, Ionomycin, and GolgiPlug and stained for surface and intracellular antigens. Superimposed bar charts illustrate GM-CSF, IFN-γ, IL-17, TNF, IL-4, and IL-10 expression by gated YFP^+^ and YFP^–^ CD4^+^ T cells from **(A)** naïve and **(B)** EAE mice. Results are expressed as the mean ± SEM; naïve (total n = 7) and EAE-immunized (total n = 8) from two independent experiments. Statistical analysis was performed using Student’s t-test; *P<0.05.

**Figure S9. The cytokine expression by YFP^+^ and YFP**^–^ **CD8^+^ T cells.** Mononuclear cells were isolated from the blood, LNs, spleen, liver, lung, SI, and CNS of 2-3-month-old male and female naïve and EAE *Gr/fr* mice. Cells were stimulated with PMA, Ionomycin, and GolgiPlug and stained for surface and intracellular antigens. Expression of GM-CSF, IFN-γ, IL-17, TNF, IL-4, and IL-10 by gated YFP^+^ and YFP^–^ CD8^+^ T cells from **(A)** naïve and **(B)** EAE mice at the peak of clinical disease severity is shown using superimposed bar charts. Results are expressed as the mean ± SEM; naïve mice (total n = 7) and mice with EAE (total n = 8) from two independent experiments. Statistical analysis was performed using Student’s t-test; *P<0.05.

**Figure S10. GM-CSF and YFP expression during Th cell polarization.** Naïve YFP^−^CD4⁺ T cells (CD4⁺CD25^−^CD62L⁺CD44^lo^YFP^−^) were sorted from the spleens of naïve 2-3-month-old male and female *Gr/fr* mice and co-cultured with T cell-depleted splenocytes at a ratio of 1:4 in the presence of soluble anti-CD3/CD28 mAbs (3 µg/ml) under ThGM and Th1 differentiation conditions; ThGM: anti-IFN-γ (10 μg/ml), anti-IL-12 (10 μg/ml), anti-IL-4 (5 μg/ml) mAbs, and Th1: IL-12 (20 ng/ml). Cells were harvested every 24h over a 5-day period, then stimulated with PMA, Ionomycin, and GolgiPlug, followed by staining for CD4, GM-CSF, and IFN-γ. **(A)** Flow cytometry plots illustrate gating for GM-CSF, IFN-γ, and YFP expression in gated CD4⁺ T cells after 72h stimulation. **(B)** GM-CSF, IFN-γ, and YFP expression in gated CD4⁺ T cells derived from naïve CD4⁺YFP^−^ T cells over time. Data is presented as mean ± SEM from a single experiment (n=3).

**Table S1. Anti-mouse flow cytometry antibodies.**

| **Antigen** | **Fluorochrome** | **Clone** | **Supplier** | **Category** |
| --- | --- | --- | --- | --- |
| CD3 | APC/Cy7 | 17A2 | Biolegend | Surface |
| CD3 | AF700 | 17A2 | Biolegend | Surface |
| CD4 | AF700 | GK1.5 | Biolegend | Surface |
| CD4 | BV711 | RM4-5 | Biolegend | Surface |
| CD4 | BV650 | GK1.5 | Biolegend | Surface |
| CD8 | PE/Cy7 | 53-6.7 | Biolegend | Surface |
| CD8 | BV711 | 53-6.7 | Biolegend | Surface |
| CD8 | APC/Cy7 | 53-6.7 | Biolegend | Surface |
| CD19 | PE | 6D5 | Biolegend | Surface |
| CD25 | BV711 | PC61 | Biolegend | Surface |
| CD25 | AF700 | PC61 | Biolegend | Surface |
| CD27 | BV685 | LG.3A10 | Biolegend | Surface |
| CD44 | BV650 | IM7 | Biolegend | Surface |
| CD44 | PE/Cy7 | IM7 | Biolegend | Surface |
| CD45 | AF700 | 30-F11 | Biolegend | Surface |
| CD45 | BV650 | 30-F11 | Biolegend | Surface |
| CD62L | APC | MEL-14 | Biolegend | Surface |
| CD62L | PE/Cy5 | MEL-14 | Biolegend | Surface |
| CD95 | BV605 | SA367H8 | Biolegend | Surface |
| CD122 | PE | 5H4 | Biolegend | Surface |
| CD127 | APC/Cy7 | A7R34 | Biolegend | Surface |
| Sca-1 | BV711 | D7 | Biolegend | Surface |
| TCR γδ | APC | QA20A46 | Biolegend | Surface |
| NK-1.1 | BV785 | PK136 | Biolegend | Surface |
| CXCR6 | PE/Dazzle™ 594 | SA051D1 | Biolegend | Surface |
| CD11b | BV785 | M1/70 | Biolegend | Surface |
| CD11c | PE | N418 | Biolegend | Surface |
| Ly6C | BV650 | HK1.4 | Biolegend | Surface |
| Ly6G | BV711 | 1A8 | Biolegend | Surface |
| F4/80 | PE/Cy7 | BM8 | Biolegend | Surface |
| MHC-II | APC/Cy7 | M5/114.15.2 | Biolegend | Surface |
| IFN-γ | PE/Cy7 | XMG1.2 | Biolegend | Intercellular |
| TNF | APC/Cy7 | MP6-XT22 | Biolegend | Intercellular |
| GM-CSF | PE/Dazzle™ 594 | MP1-22E9 | Biolegend | Intercellular |
| IL-2 | PE | JES6-5H4 | BD Biosciences | Intercellular |
| IL-4 | APC | 11B11 | Biolegend | Intercellular |
| IL-10 | PE | JES5-16E3 | Biolegend | Intercellular |
| IL-17 | BV785 | TC11-18H10.1 | Biolegend | Intercellular |
| Foxp3 | PE | FJK-16S | eBioscience | Intercellular |
| IL; Interleukin, IFN; Interferon, TNF; Tumor necrosis factor, GM-CSF; Granulocyte-macrophage colony-stimulating factor, Foxp3; Forkhead box P3, Sca-1; Stem cell antigen-1, CXCR; CXC chemokine receptor, MHC class II; Major Histocompatibility Complex class II. | | | | |

**Table S2, refer to Figure 2.** Frequency of immune cell subsets among CD45^hi^YFP⁺ cells in various organs of naïve and EAE mice at disease peak.

|  |  | Blood | BM | SI | Liver | LN | Spleen | Lung | Thymus | CNS | *P-value* |
| --- | --- | --- | --- | --- | --- | --- | --- | --- | --- | --- | --- |
| CD3^+^CD4^+^ (%) | Naive | 27.1±9.3 | 4.6±1.6 | 4.0±1.7 | 21.4±8.2 | 59.4±4.6 | 27.8±4.7 | 17.9±2.9 | 51.4±10.4 | 18.0±6.0 | ***<0.001*** |
|  | EAE | 25.8±3.7 | 3.3±0.8 | 3.8±0.9 | 22.1±1.4 | 54.7±8.7 | 31.1±6.7 | 30.8±4.0 | 49.6±8.9 | 47.1±11.5 | ***<0.001*** |
|  | ***P-value*** | ***0.74*** | ***0.15*** | ***0.78*** | ***0.80*** | ***0.12*** | ***0.20*** | ***<0.001*** | ***0.75*** | ***0.01*** |  |
| CD3^+^CD8^+^(%) | Naive | 9.3±3.9 | 17.2±7.4 | 39.3±5.6 | 9.6±2.6 | 11.8±2.5 | 10.8±4.0 | 7.4±1.9 | 1.7±0.5 | 14.4±3.4 | ***<0.001*** |
|  | EAE | 12.5±4.7 | 25.8±2.5 | 49.4±8.4 | 9.4±1.2 | 12.7±2.3 | 12.9±3.0 | 8.7±2.5 | 4.7±2.3 | 8.7±3.3 | ***<0.001*** |
|  | ***P-value*** | ***0.12*** | ***0.04*** | ***0.006*** | ***0.87*** | ***0.42*** | ***0.21*** | ***0.20*** | ***<0.001*** | ***0.06*** |  |
| CD3^+^CD4^-^CD8^-^ (%) | Naive | 2.2±0.6 | 23.3±3.0 | 3.8±1.9 | 10.8±3.0 | 2.9±1.0 | 4.3±0.7 | 6.3±2.1 | 5.8±2.9 | 12.6±2.26 | ***<0.001*** |
|  | EAE | 3.6±0.8 | 20.6±3.9 | 5.2±1.8 | 12.6±1.3 | 4.3±2.4 | 4.5±0.8 | 8.5±1.9 | 18.2±4.0 | 12.3±3.8 | ***<0.001*** |
|  | ***P-value*** | ***0.0004*** | ***0.1639*** | ***0.1477*** | ***0.14*** | ***0.08*** | ***0.61*** | ***0.02*** | ***<0.001*** | ***0.18*** |  |
| CD3^+^NK1.1^+^ (%) | Naive | 0.5±0.6 | 7.9±6.5 | 1.2±1.5 | 3.0±3.2 | 0.6±0.4 | 0.8±0.8 | 0.5±0.4 | 1.4±0.7 | 0.3±0.05 | ***<0.001*** |
|  | EAE | 0.2±0.1 | 0.9±0.9 | 1.3±0.8 | 0.6±0.9 | 0.2±0.07 | 0.2±0.08 | 0.2±0.1 | 1.7±1.3 | 0.24±0.08 | ***<0.001*** |
|  | ***P-value*** | ***0.32*** | ***0.05*** | ***0.88*** | ***0.05*** | ***0.06*** | ***0.04*** | ***0.06*** | ***0.63*** | ***0.18*** |  |
| CD3+TCRγδ+ (%) | Naive | 4.4±3.0 | 9.3±5.1 | 40.6±6.0 | 13.1±3.4 | 8.0±2.7 | 5.5±2.8 | 19.7±4.7 | 20.7±13.4 | 1.21±0.82 | ***<0.001*** |
|  | EAE | 4.3±1.7 | 2.5±0.3 | 25.1±7.6 | 5.5±1.2 | 12.0±8.3 | 5.1±2.5 | 24.4±1.9 | 5.8±1.5 | 8.6±4.9 | ***<0.001*** |
|  | ***P-value*** | ***0.93*** | ***0.02*** | ***<0.001*** | ***<0.001*** | ***0.12*** | ***0.75*** | ***0.01*** | ***0.04*** | ***0.07*** |  |
| CD3-NK1.1+ (%) | Naive | 2.9±1.4 | 0.8±0.3 | 1.1±1.5 | 2.5±1.4 | 1.7±0.8 | 1.5±0.7 | 1.5±1.4 | 6.0±2.7 | 2.24±5.32 | ***<0.001*** |
|  | EAE | 3.6±1.0 | 0.5±0.1 | 0.6±0.3 | 11.5±9.6 | 4.4±2.9 | 0.9±0.02 | 5.9±4.1 | 4.3±4.7 | 5.3±1.6 | ***0.01*** |
|  | ***P-value*** | ***0.39*** | ***0.38*** | ***0.45*** | ***0.07*** | ***0.07*** | ***0.32*** | ***0.05*** | ***0.37*** | ***0.06*** |  |
| CD19+ (%) | Naive | 1.1±0.8 | 0.4±0.2 | 0.1±0.2 | 0.4±0.1 | 0.5±0.4 | 3.1±1.1 | 0.3±0.2 | 0.5±0.2 | 0.31±0.26 | ***<0.001*** |
|  | EAE | 0.7±0.6 | 0.06±0.04 | 0.04±0.03 | 0.1±0.07 | 1.0±0.9 | 2.2±1.8 | 0.3±0.2 | 0.4±0.1 | 0.09±0.08 | ***<0.001*** |
|  | ***P-value*** | ***0.28*** | ***0.008*** | ***0.48*** | ***<0.001*** | ***0.10*** | ***0.18*** | ***0.67*** | ***0.75*** | ***0.04*** |  |
| CD11b+ (%) | Naive | 46.1±8.3 | 20.9±6.3 | 5.3±2.7 | 23.1±4.8 | 7.1±2.8 | 41.8±6.0 | 38.7±6.2 | 2.2±1.1 | 23.6±2.1 | ***<0.001*** |
|  | EAE | 41.8±9.8 | 15.6±5.5 | 5.6±2.4 | 21.7±2.1 | 5.5±2.4 | 33.7±8.5 | 22.5±4.4 | 1.7±0.6 | 16.0±1.9 | ***<0.001*** |
|  | ***P-value*** | ***0.30*** | ***0.15*** | ***0.84*** | ***0.46*** | ***0.21*** | ***0.02*** | ***<0.001*** | ***0.50*** | ***<0.001*** |  |
| *Data are presented as mean ± SD from three independent experiments in naïve mice (total n = 13) and two independent experiments in EAE mice (total n = 8). Statistical analysis was performed using ANOVA for comparisons of immune cell populations across organs, and multiple t-tests for comparisons between naïve and EAE mice. Abbreviations: LN – lymph nodes; BM – bone marrow; SI – small intestine; CNS – central nervous system.* | | | | | | | | | | | |
